# NIHSS and Large Artery Atherosclerosis Affect Efficacy of Dual Antiplatelet Therapy Versus Alteplase for Minor Stroke

**DOI:** 10.1002/brb3.71813

**Published:** 2026-10-05

**Authors:** Yu Cui, Zi‐Ang Li, Chao He, Hui‐Sheng Chen

**Affiliations:** ^1^ Department of Neurology General Hospital of Northern Theater Command Shenyang China

**Keywords:** antiplatelet, intravenous thrombolysis, Large artery atherosclerosis, Minor stroke, NIHSS score

## Abstract

**Background:**

Dual antiplatelet therapy (DAPT) was noninferior to alteplase in nondisabling minor stroke (NIHSS 0 to 5). Given potential benefit of DAPT in minor stroke with large artery atherosclerosis (LAA) and of alteplase in stroke with NIHSS 4–5, we hypothesize that neurological deficit and LAA may affect efficacy of DAPT versus alteplase in minor stroke.

**Methods:**

This analysis included patients from the ARAMIS trial with minor stroke who underwent vessel imaging assessment. Participants were stratified into NIHSS 0–3 with LAA, NIHSS 0–3 with Non‐LAA, NIHSS 4–5 with LAA, and NIHSS 4–5 with Non‐LAA. Outcomes were compared between those receiving DAPT versus alteplase. Primary outcome was an excellent functional outcome (mRS 0 to 1) at 90 days. Safety outcomes included symptomatic intracranial hemorrhage and bleeding events. Primary analysis employed adjusted models, with sensitivity analysis conducted using inverse probability of treatment weighting.

**Results:**

Among 478 included patients, including 388 in NIHSS 0–3 group and 90 in NIHSS 4–5 group. In NIHSS 0–3 group, a numerically higher rates of excellent functional outcome was found in patients with LAA who received DAPT versus alteplase (97.3% vs. 85.1%; adjusted OR, 6.45 [0.68 to 60.81]; *p* = 0.10), which was obviously attenuated in patients with Non‐LAA (96.5% vs. 94.4%; adjusted OR, 1.67 [0.50 to 5.59]; *p* = 0.41). This phenomenon was not observed in NIHSS 4–5 group, and the test for interaction was not statistically significant (*p*‐interaction = 0.36). Notably, sensitivity analysis revealed that DAPT significantly increased the likelihood of excellent functional outcome in the NIHSS 0–3 with LAA subgroup (adjusted OR, 6.49 [1.54 to 27.39]; *p* = 0.01), but not in NIHSS 0–3 with Non‐LAA subgroup (adjusted OR, 2.51 [0.58 to 10.90]; *p* = 0.22). Safety outcomes were comparable across treatments.

**Conclusions:**

Neurological deficit and LAA may affect efficacy of DAPT versus alteplase in acute minor nondisabling stroke. In patients with NIHSS scores of 0 to 3 due to LAA, DAPT may be associated with numerically higher odds of 90‐day functional outcomes compared to alteplase. Given the exploratory nature of this subgroup analysis and no formal adjustment for multiple comparisons was applied, these results should be interpreted as hypothesis‐generating and require confirmation.

## Graphical abstract

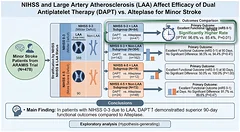

## 1 Introduction

Intravenous thrombolysis remains a standard treatment for acute ischemic stroke within 4.5 h of symptom onset according to current guideline (Powers et al. 2019). However, its necessity in patients with nondisabling minor stroke is debated. The Antiplatelet Versus R‐tPA for Acute Mild Ischemic Stroke (ARAMIS) trial first established the non‐inferiority of dual antiplatelet therapy (DAPT) to intravenous alteplase in this population (Chen et al. 2023). More recently, a secondary analysis of the Tenecteplase versus Standard of Care for Minor Ischaemic Stroke with Proven Occlusion (TEMPO‐2) trial indicated that even in patients with disabling minor stroke and intracranial occlusion, intravenous thrombolysis provided no significant clinical benefit (Zhang et al. 2025). Given that intravenous thrombolysis appears to offer limited advantage in both disabling and nondisabling minor strokes, antiplatelet‐based standard treatments such as DAPT are increasingly considered potential therapeutic alternatives. Nevertheless, a meta‐analysis found that DAPT did not significantly improve the rate of excellent functional outcomes compared with thrombolysis (Zhang et al. 2024), raising the critical question of which patient subgroups might derive greater benefit from antiplatelet‐based regimens.

A national registry study in China suggested that patients with minor stroke and a National Institutes of Health Stroke Scale (NIHSS) score greater than 3 may derive benefit from intravenous alteplase (Yin et al. 2025). Similarly, a recent prespecified post hoc analysis of the ARAMIS trial reported a trend toward more favorable 90‐day functional outcomes with dual antiplatelet therapy (DAPT) in patients with minor stroke (NIHSS score 0–3) (Fei et al. 2025). Although interaction analysis indicated that NIHSS score influenced treatment prognosis, no statistically significant benefit was detected within the NIHSS score 0–3 subgroup itself, highlighting the need to identify specific populations within this score range. Notably, patients with large‐artery atherosclerosis (LAA) as the stroke mechanism are at higher risk of early neurological deterioration (END) and tend to have lower rates of favorable functional outcomes (Yu et al. 2020; Park et al. 2020). The evidence from CHANCE (Clopidogrel in High‐Risk Patients with Acute Non‐disabling Cerebrovascular Events) and INSPIRES (Intensive Statin and Antiplatelet Therapy for Acute High Risk Intracranial or Extracranial Atherosclerosis) suggested that DAPT favored minor stroke patients with LAA (Liu et al. 2015; Gao et al. 2023). Furthermore, our previous work indicated the greater benefit of DAPT with clopidogrel and aspirin in mitigating END in LAA‐related stroke (Cui et al. 2024). In this context, we hypothesize that neurological deficit and LAA may affect efficacy of DAPT versus alteplase in acute minor stroke.

Based on this rationale, we conducted an exploratory analysis using data from patients in the ARAMIS trial who underwent vessel imaging to evaluate whether NIHSS (0–3 vs. 4–5) and LAA may affect efficacy of DAPT versus alteplase in acute minor nondisabling stroke.

## 2 Methods

The datasets generated and/or analyzed during the current study are available from the corresponding author upon reasonable request.

### 2.1 Study Design, Participants, and Procedure

This study constitutes a secondary analysis of the ARAMIS trial, the detailed design of which has been previously published (Wang et al. 2019). We included patients from the as‐treated analysis set of ARAMIS who were diagnosed with acute nondisabling ischemic stroke with NIHSS score 0–5, underwent responsible vessel examination, and completed the 90‐day follow‐up. The full inclusion and exclusion criteria have been described in the primary study (Wang et al. 2019).

Based on the degree of neurological deficits and vessel stenosis, eligible patients were categorized into four subgroups: NIHSS 0–3 with LAA, NIHSS 0–3 with Non‐LAA, NIHSS 4–5 with LAA, and NIHSS 4–5 with Non‐LAA. The adjudication of LAA was based on vessel ultrasonography or imaging examination by MRA/CTA, which was likely to account for the clinical presentation and acute cerebral infarction, by clinical physicians who were not blinded to treatment assignment. The stenosis of a major intracranial or extracranial artery should achieve at least 50% in the examination. Within each subgroup, patients were further stratified according to the treatment they actually received. Those in the DAPT group received a loading dose of clopidogrel (300 mg) and aspirin (100 mg) on day 1. Patients in the alteplase group received intravenous thrombolysis at a standard dose of 0.9 mg/kg (maximum 90 mg). From day 2 to day 90, all patients in both groups received standard secondary prevention care in accordance with guideline (Powers et al. 2019).

All data were prospectively recorded using an electronic data capture system (Shanghai Meisi Medical Technology Co, Ltd). Collected information encompassed neurological status at admission and 24 h post‐randomization, baseline patient characteristics at randomization, and follow‐up outcomes at 90 days.

### 2.2 Outcomes

The primary outcome was an excellent functional outcome at 90 days, defined as a modified Rankin Scale (mRS) score of 0 or 1. Secondary efficacy outcomes encompassed a favorable functional outcome (mRS 0–2) at 90 days, ordinal shift in the mRS distribution at 90 days, END at 24 h (an increase of ≥4 points in the NIHSS score from baseline), and early neurological improvement (ENI) at 24 h (a decrease of ≥2 points in the NIHSS score from baseline).

Safety outcomes were symptomatic intracranial hemorrhage (SICH) and bleeding events. SICH was defined according to established criteria as the presence of any intracranial hemorrhage on neuroimaging, accompanied by neurological deterioration (an increase of ≥4 points in the NIHSS score) that was attributable to the hemorrhage (Hacke et al. 2008).

### 2.3 Statistical Analysis

To assess potential selection bias, we compared the baseline characteristics of included versus excluded patients in the current study. Furthermore, given the potential for imbalances between treatment groups after stratification into subgroups, the primary analyses were adjusted for covariates.

Baseline characteristics were summarized using medians with interquartile ranges (IQR) for continuous variables and frequencies with percentages for categorical variables. For outcome comparisons, we reported the absolute number of events (percentages) along with odds ratios (ORs) and their corresponding 95% confidence intervals (CIs).

Baseline characteristics were compared across subgroups and between treatment groups. The association between treatments and outcomes was evaluated within each subgroup separately. Binary logistic regression models were used to calculate adjusted ORs for excellent functional outcome, favorable functional outcome, ordinal shift in mRS scores, END, ENI, and safety outcomes. Two‐sided 95% CIs and adjusted *p*‐values were reported for all treatment effect estimates. Covariates that showed a between‐group difference at *p* <0.1 and those prespecified adjusted in the ARAMIS trial (including age, sex, history of diabetes, NIHSS score at admission, time from onset of symptom to intravenous thrombolysis or dual antiplatelet therapy, and location of responsible vessel) were included in the adjusted models. Treatment‐by‐subgroup interactions for all outcomes were formally tested.

Given the small sample size in certain subgroups (especially NIHSS 4–5 with LAA) and the observed baseline imbalances between groups, standard multivariable adjustment might be insufficient. We therefore used inverse probability of treatment weighting (IPTW) as a primary sensitivity analysis to create a dataset in which the distribution of measured covariates was balanced between groups, thereby reducing confounding and improving the stability of estimates.

A logistic regression model was used to estimate the probability of DAPT versus alteplase. The dependent variable was the subgroup, and the independent variables included the same covariates with above adjusted analysis. We calculated inverse probability weights for the average treatment effect. Specifically, weights were calculated as 1/PS for patients treated with DAPT and 1/(1−PS) for patients treated with alteplase. We calculated all standardized mean differences for baseline covariates between groups to identify the inter‐group balance after IPTW. After weighting subgroups, the primary outcome was reanalyzed in this new dataset.

All analyses were exploratory, and reported *p*‐values are nominal. A two‐sided *p*‐value < 0.05 was considered statistically significant. All statistical analyses were performed using IBM SPSS Statistics (Version 26.0).

## 3 Results

### 3.1 Participants

As shown in Figure 1, the final analysis comprised 478 patients, stratified as follows: 84 in the NIHSS 0–3 with LAA subgroup (DAPT *n* = 37; alteplase *n* = 47), 304 in the NIHSS 0–3 with Non‐LAA subgroup (DAPT *n* = 142; alteplase *n* = 162), 18 in the NIHSS 4–5 with LAA subgroup (DAPT *n* = 6; alteplase *n* = 12), and 72 in the NIHSS 4–5 with Non‐LAA subgroup (DAPT *n* = 24; alteplase *n* = 48).

**FIGURE 1:**
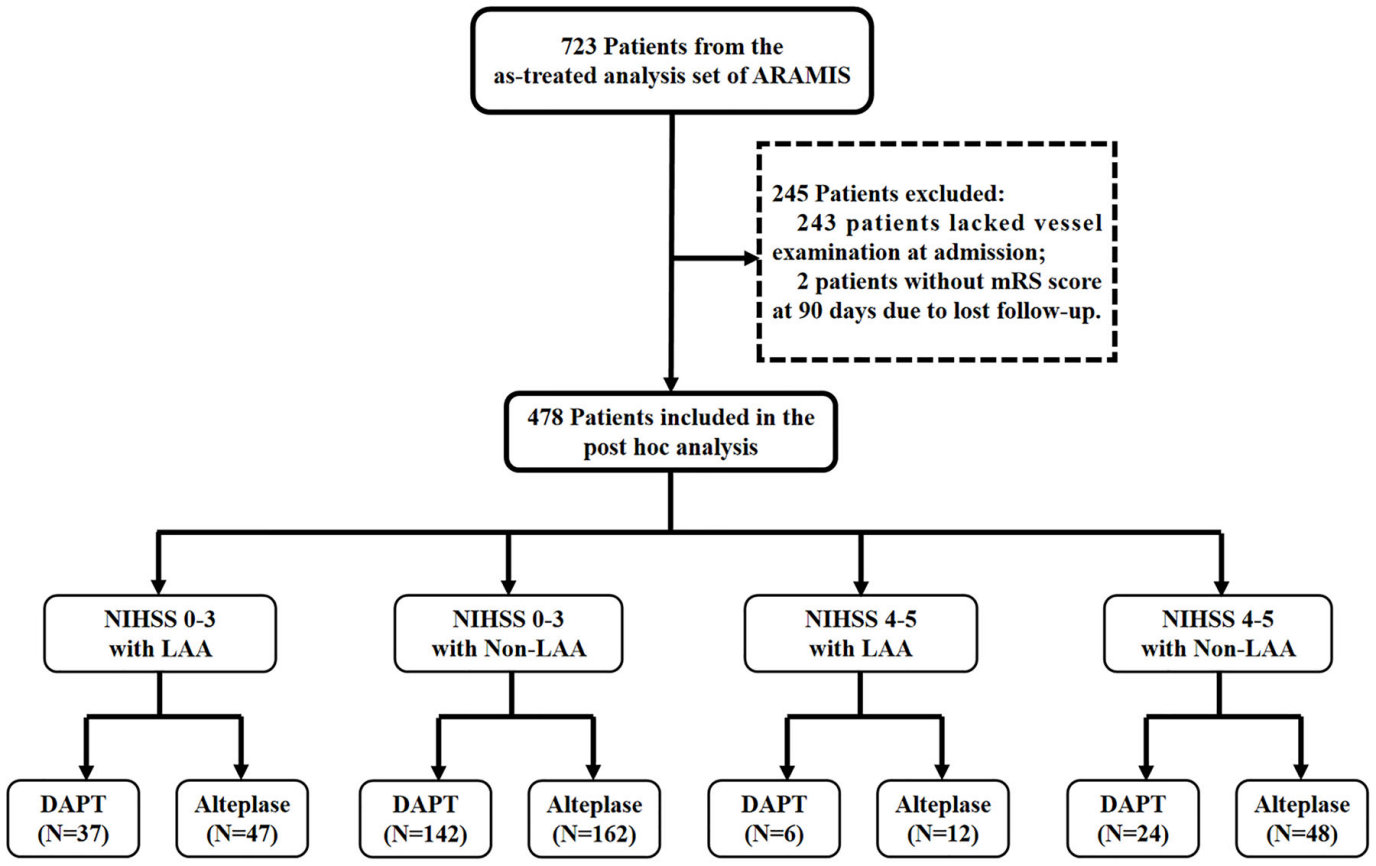
Flow chart. ARAMIS indicates Antiplatelet Versus R‐tPA for Acute Mild Ischemic Stroke; DAPT, dual antiplatelet therapy; LAA, large‐artery atherosclerosis; mRS, modified Rankin Scale; NIHSS, National Institutes of Health Stroke Scale.

A comparison of baseline characteristics between included and excluded patients in the current study was presented in Table S1. The baseline characteristics among subgroups showed some imbalances including gender, current drinker, history of diabetes, systolic blood pressure at admission, fast blood glucose at admission, duration of hospitalization, and presumed stroke etiology (Table S2). Furthermore, the full details of baseline characteristics for each treatment group were provided in Tables S3‐S6.

### 3.2 Outcomes

In the primary analysis of patients with NIHSS scores of 0–3, DAPT was associated with a numerically higher increase in proportion of excellent functional outcomes at 90 days compared to alteplase in the LAA (97.3% vs. 85.1%; adjusted OR, 6.45 [95% CI, 0.68 to 60.81]; *p* = 0.10), vs. Non‐LAA (96.5% vs. 94.4%; adjusted OR, 1.67 [95% CI, 0.50 to 5.59]; *p* = 0.41) subgroups (Table 1). Conversely, among patients with NIHSS scores of 4–5, a slightly numerical advantage for DAPT was observed only in the Non‐LAA subgroup (91.7% vs. 89.6%; adjusted OR, 2.20 [95% CI, 0.26 to 18.38]; *p* = 0.47), but not in the LAA subgroup (50.0% vs. 100.0%; adjusted *p* > 0.99) (Table 2). However, tests for interaction between treatment effect and etiological subgroup were not statistically significant in either the NIHSS 0–3 (*p*‐interaction = 0.36) or NIHSS 4–5 (*p*‐interaction>0.99) patients.

**TABLE 1:** Comparison outcomes in patients with NIHSS 0–3.

Outcomes	Subgroups	No.(%) of events		Unadjusted		Adjusted ^a^		*p*_int_ ^e^ value
		DAPT	Alteplase	OR (95% CI)	*p* value	OR (95% CI)	*p* value	
**Primary outcome**								
mRS scoring 0 to 1 at 90 days	LAA ^f^	36 (97.3)	40 (85.1)	6.30 (0.74 to 53.71)	0.09	6.45 (0.68 to 60.81)	0.10	0.36
	Non‐LAA	137 (96.5)	153 (94.4)	1.61 (0.53 to 4.93)	0.40	1.67 (0.50 to 5.59)	0.41	
**Secondary outcomes**								
mRS scoring 0 to 2 at 90 days	LAA	37 (100.0)	41 (87.2)	NA (0.00 to NA)	>0.99	NA (0.00 to NA)	>0.99	>0.99
	Non‐LAA	139 (97.9)	158 (97.5)	1.17 (0.26 to 5.33)	0.84	1.18 (0.23 to 6.10)	0.85	
mRS score distribution at 90 days	LAA	NA	NA	1.85 (0.66 to 5.20)	0.24	2.48 (0.77 to 7.95)	0.13	0.61
	Non‐LAA	NA	NA	1.29 (0.71 to 2.32)	0.40	1.29 (0.69 to 2.43)	0.43	
END ^b^ within 24 h	LAA	2 (5.4)	6 (12.8)	0.39 (0.07 to 2.06)	0.27	0.50 (0.11 to 2.24)	0.36	>0.99
	Non‐LAA	0 (0.0)	8 (4.9)	0.00 (0.00 to NA)	>0.99	0.00 (0.00 to NA)	>0.99	
ENI ^c^ within 24 h	LAA	1 (2.7)	6 (12.8)	0.19 (0.02 to 1.65)	0.13	0.05 (0.00 to 1.07)	0.06	0.23
	Non‐LAA	15 (10.6)	26 (16.0)	0.62 (0.31 to 1.22)	0.17	1.05 (0.47 to 2.35)	0.90	
**Safety outcomes**								
sICH ^d^	LAA	0 (0.0)	1 (2.1)	0.00 (0.00 to NA)	>0.99	0.00 (0.00 to NA)	>0.99	>0.99
	Non‐LAA	0 (0.0)	1 (0.6)	0.00 (0.00 to NA)	>0.99	0.00 (0.00 to NA)	>0.99	
Bleeding events	LAA	0 (0.0)	5 (10.6)	0.00 (0.00 to NA)	>0.99	0.00 (0.00 to NA)	>0.99	>0.99
	Non‐LAA	1 (0.7)	6 (3.7)	0.18 (0.02 to 1.55)	0.12	0.22 (0.02 to 2.07)	0.18	
**IPTW sensitivity analysis**								
mRS scoring 0 to 1 at 90 days	LAA (*N* = 170)	85/88 (96.6)	70/82 (85.4)	5.66 (1.43 to 22.40)	0.01	6.49 (1.54 to 27.39)	0.01	0.26
	Non‐LAA (*N* = 375)	69/71 (97.2)	286/304 (94.1)	1.84 (0.46 to 7.3)	0.39	2.51 (0.58 to 10.90)	0.22	

Abbreviations: CI, confidence interval; DAPT, dual antiplatelet therapy; END, early neurological deterioration; ENI, early neurological improvement; IPTW, inverse probability of treatment weighting; LAA, large artery atherosclerosis; mRS, modified Rankin Scale; N/A, not applicable; NIHSS, National Institute of Health Stroke Scale; OR, odds ratio; sICH, symptomatic intracerebral hemorrhage.

^a^ Adjusted for covariates compared between DAPT and alteplase treatments with *p* value <0.1 in each subgroup in Tables S3 and S4, and those prespecified adjusted in the ARAMIS trial (including age, sex, history of diabetes, NIHSS score at admission, time from onset of symptom to intravenous thrombolysis or dual antiplatelet therapy, and location of responsible vessel).

^b^ END was defined as an increase between baseline and 24 h of 4 points on the NIHSS score, but not as a result of cerebral hemorrhage.

^c^ ENI was defined as a decrease between baseline and 24 h of 2 points on the NIHSS score.

^d^ SICH was defined as any evidence of bleeding on head CT associated with neurological deterioration (NIHSS ≥ 4‐point increase).

^e^ *p* value for interaction was calculated by adjusting covariates compared between subgroups with *p* value < 0.1 in Table S8, and those prespecified adjusted in the ARAMIS trial (including age, sex, history of diabetes, NIHSS score at admission, time from onset of symptom to intravenous thrombolysis or dual antiplatelet therapy, and location of responsible vessel).

^f^ The small sample size in the LAA subgroups, means that the non‐significant results in the primary analysis may represent a Type II error. Therefore, our findings should be considered exploratory and hypothesis‐generating, mandating validation in a dedicated, adequately powered prospective cohort.

**TABLE 2:** Comparison of outcomes in patients with NIHSS 4–5.

Outcomes	Subgroups	No.(%) of events		Unadjusted		Adjusted ^a^		*p*_int_ ^e^ value
		DAPT	Alteplase	OR (95% CI)	*p* value	OR (95% CI)	*p* value	
**Primary outcome**								
mRS scoring 0 to 1 at 90 days	LAA ^f^	3 (50.0)	12 (100.0)	0.00 (0.00 to NA)	>0.99	0.00 (0.00 to NA)	>0.99	>0.99
	Non‐LAA	22 (91.7)	43 (89.6)	1.28 (0.23 to 7.13)	0.78	2.20 (0.26 to 18.38)	0.47	
**Secondary outcomes**								
mRS scoring 0 to 2 at 90 days	LAA	3 (50.0)	12 (100.0)	0.00 (0.00 to NA)	>0.99	0.00 (0.00 to NA)	>0.99	>0.99
	Non‐LAA	23 (95.8)	47 (97.9)	0.49 (0.03 to 8.18)	0.62	0.29 (0.00 to 80.33)	0.67	
mRS score distribution at 90 days	LAA	NA	NA	NA (0.00 to NA)	>0.99	NA (0.00 to NA)	>0.99	>0.99
	Non‐LAA	NA	NA	0.52 (0.21 to 1.33)	0.17	0.71 (0.22 to 2.27)	0.56	
END ^b^ within 24 h	LAA	1 (16.7)	1 (8.3)	2.20 (0.11 to 42.74)	0.60	0.00 (0.00 to NA)	>0.99	>0.99
	Non‐LAA	0 (0.0)	2 (4.2)	0.00 (0.00 to NA)	>0.99	0.00 (0.00 to NA)	>0.99	
ENI ^c^ within 24 h	LAA	3 (50.0)	5 (41.7)	1.40 (0.20 to 10.03)	0.74	NA (NA to NA)	NA	0.49
	Non‐LAA	7 (29.2)	15 (31.3)	0.91 (0.31 to 2.64)	0.86	1.54 (0.36 to 6.61)	0.56	
**Safety outcomes**								
sICH ^d^	LAA	0 (0.0)	0 (0.0)	NA (NA to NA)	NA	NA (NA to NA)	NA	NA
	Non‐LAA	0 (0.0)	0 (0.0)	NA (NA to NA)	NA	NA (NA to NA)	NA	
Bleeding events	LAA	0 (0.0)	0 (0.0)	NA (NA to NA)	NA	NA (NA to NA)	NA	NA
	Non‐LAA	0 (0.0)	8 (16.7)	0.00 (0.00 to NA)	>0.99	0.00 (0.00 to NA)	>0.99	
**IPTW sensitivity analysis**								
mRS scoring 0 to 1 at 90 days	LAA (*N* = 29)	3/13 (23.1%)	16/16 (100.0%)	0.00 (0.00 to NA)	>0.99	0.00 (0.00 to NA)	>0.99	>0.99
	Non‐LAA (*N* = 136)	58/63 (92.1%)	66/73 (90.4%)	1.24 (0.38 to 4.04)	0.72	2.67 (0.58 to 12.38)	0.21	

Abbreviations: CI, confidence interval; DAPT, dual antiplatelet therapy; END, early neurological deterioration; ENI, early neurological improvement; IPTW, inverse probability of treatment weighting; LAA, large artery atherosclerosis; mRS, modified Rankin Scale; N/A, not applicable; NIHSS, National Institute of Health Stroke Scale; OR, odds ratio; sICH, symptomatic intracerebral hemorrhage.

^a^ Adjusted for covariates compared between DAPT and alteplase treatments with *p* value <0.1 in each subgroup in Tables S5 and S6, and those prespecified adjusted in the ARAMIS trial (including age, sex, history of diabetes, NIHSS score at admission, time from onset of symptom to intravenous thrombolysis or dual antiplatelet therapy, and location of responsible vessel).

^b^ END was defined as an increase between baseline and 24 h of 4 points on the NIHSS score, but not as a result of cerebral hemorrhage.

^c^ ENI was defined as a decrease between baseline and 24 h of 2 points on the NIHSS score. In the LAA subgroup, the number of events was too small to support stable multivariable logistic regression. Adjusted ORs are therefore not reported for this subgroup; only crude event proportions are presented.

^d^ SICH was defined as any evidence of bleeding on head CT associated with neurological deterioration (NIHSS ≥ 4‐point increase).

^e^ *p* value for interaction was calculated by adjusting covariates compared between subgroups with *p* value < 0.1 in Table S9, and those prespecified adjusted in the ARAMIS trial (including age, sex, history of diabetes, NIHSS score at admission, time from onset of symptom to intravenous thrombolysis or dual antiplatelet therapy, and location of responsible vessel).

^f^ The small sample size in the LAA subgroups, means that the non‐significant results in the primary analysis may represent a Type II error. Therefore, our findings should be considered exploratory and hypothesis‐generating, mandating validation in a dedicated, adequately powered prospective cohort.

The distribution of mRS scores at 90 days was presented in Figure 2. Regarding secondary and safety outcomes, no significant differences were found between the treatment groups. Furthermore, no significant interaction between treatment effect and etiological subgroup was identified in either the NIHSS 0–3 or the NIHSS 4–5 patient groups (Tables 1 and 2).

**FIGURE 2:**
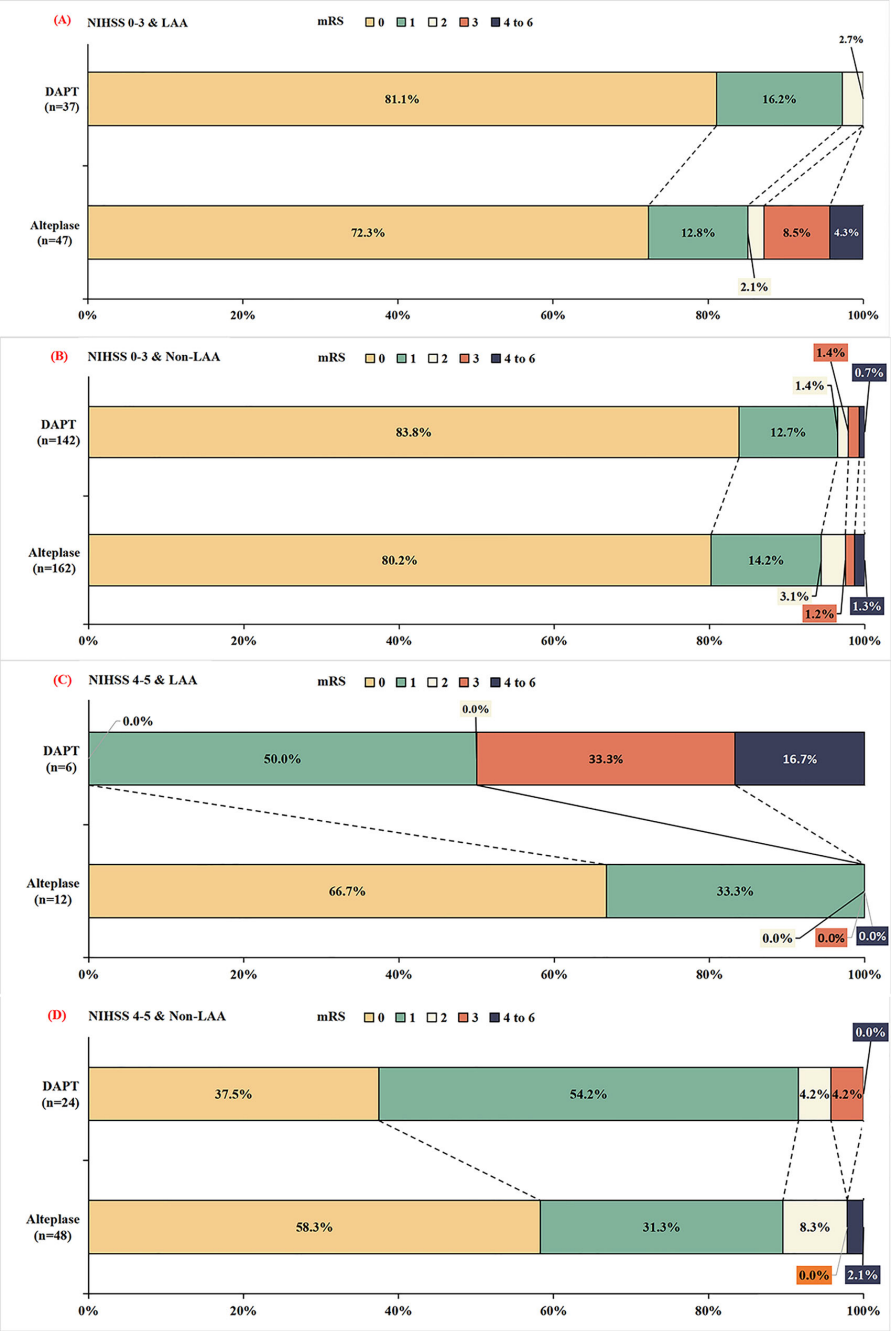
Distribution of 90‐day mRS score in subgroups. (A) Patients with NIHSS scoring 0–3 at admission and LAA etiology. (B) Patients with NIHSS scoring 0–3 at admission and Non‐LAA etiology. (C) Patients with NIHSS scoring 4–5 at admission and LAA etiology. (D) Patients with NIHSS scoring 4–5 at admission and Non‐LAA etiology. DAPT indicates dual antiplatelet therapy; LAA, large artery atherosclerosis; mRS, modified Rankin Scale; NIHSS, National Institutes of Health Stroke Scale.

### 3.3 Sensitivity Analyses

Weight characteristics and Standardized mean difference of baseline characteristics between treatment groups were shown in Table S7, which indicated balance after IPTW. Notably, the sensitivity analysis using IPTW yielded distinct findings for the primary outcome (Tables 1 and 2). Among patients with NIHSS scores of 0–3, DAPT was associated with a significantly higher rate of excellent functional outcome in the LAA subgroup (96.6% vs. 85.4%; adjusted OR, 6.49 [95% CI, 1.54–27.39]; *p* = 0.01) but not in the non‐LAA subgroup (97.2% vs. 94.1%; adjusted OR, 2.51 [95% CI, 0.58–10.90]; *p* = 0.22). No statistically significant treatment‐by‐subgroup interaction was confirmed in this population (*p*‐interaction = 0.26). Conversely, among patients with NIHSS scores of 4–5, no significant treatment differences or subgroup interactions were observed (*p*‐interaction >0.99).

## 4 Discussion

In this study, our prespecified primary adjusted analysis did not demonstrate a statistically significant effect of DAPT over alteplase in any subgroup. The IPTW sensitivity analysis, which was performed as a post hoc exploratory tool to assess the robustness of our findings, showed a nominal *p*‐value below 0.05 in the NIHSS score 0–3 attributable to LAA subgroup; however, given its exploratory nature, the lack of multiplicity adjustment, and its dependence on the positive assumption, this finding should not be overinterpreted as confirmatory evidence. Instead, it should serve as a signal for future prospective validation.

As hypothesized, compared with intravenous alteplase, DAPT was associated with a greater likelihood of excellent functional outcomes at 90 days in minor stroke patients with milder neurological deficit attributed to LAA than in those with non‐LAA etiologies. The attenuated effect observed in the non‐LAA subgroup may be attributable to a higher proportion of good functional outcomes, leading to a ceiling effect. However, nearly half of the participants in the ARAMIS trial were excluded from this analysis. A substantial proportion of strokes with undetermined etiology may in fact have been caused by LAA (Tao et al. 2021), yet could not be classified as such due to the lack of comprehensive vascular imaging, possibly influencing the representativeness of the cohort.

Although the benefit of DAPT in the LAA subgroup was not statistically significant in the primary analysis, this may be due to the limited sample size. However, after applying IPTW, a significant benefit of DAPT emerged in LAA patients, accompanied by a significant interaction between stroke etiology and treatment effect. Additionally, the baseline diabetes imbalance (33.3% vs. 25%) may suggest a higher metabolic burden in the LAA subgroup, which has been associated with white matter alterations and poor outcomes in prior literature (Segura et al. 2010; Zhang et al. 2025; Xu et al. 2020). Nevertheless, since we did not collect white matter hyperintensities data, this remains an unverified, post hoc hypothesis that warrants confirmation in future studies integrating neuroimaging and comprehensive metabolic profiling. Lastly, there may be some impact on stroke subtype, severity, or outcomes from female sex (Torres‐Riera et al. 2025), which were unbalanced between groups. These may partially contribute to the current finding. While the primary adjusted analysis did not reach statistical significance, likely due to limited power, the IPTW sensitivity analysis, which effectively balanced baseline confounders, revealed a significant and robust benefit for DAPT in the NIHSS 0–3 with LAA subgroup. This suggests that the treatment effect is consistent in direction, though its magnitude should be interpreted with caution.

We postulate that the benefit of DAPT in the LAA subgroup may be attributable to its efficacy in preventing END. Consistent with previous literature (Ferrari et al. 2010), the incidence of END was higher in LAA patients than in non‐LAA patients (9.5% vs. 2.6%). The elevated risk in this population provides a therapeutic opportunity for DAPT, which acts by inhibiting platelet aggregation and subsequent vessel reocclusion‐key mechanisms underlying post‐thrombolytic END, particularly in ischemic stroke with underlying severe stenosis or occlusion (Rubiera et al. 2005; Alexandrov and Grotta 2002). Consequently, the higher END risk in LAA patients establishes a strong therapeutic indication for DAPT, a contention further validated by our previous study (Cui et al. 2024). Notably, a significant treatment‐by‐etiology interaction was observed in patients with NIHSS scores of 0–3, but not in those with scores of 4–5, suggesting that the benefit of DAPT is modulated by both stroke etiology and initial severity. This differential response may be explained by two interrelated mechanisms. First, LAA patients with mild deficits (NIHSS 0–3) are likely to have better‐developed collateral circulation (Guglielmi et al. 2019), in which setting DAPT may confer protection by preventing micro‐thrombosis within vulnerable collateral networks. By contrast, patients with severe deficits (NIHSS 4–5) tend to exhibit poorer collateral flow and more severe hypoperfusion, both established contributors to END (Furlanis et al. 2018; Hsu et al. 2015). Since DAPT primarily targets thrombotic mechanisms rather than improving perfusion, addresses thrombotic rather than hemodynamic impairment, its ability to prevent hypoperfusion‐driven END may be limited. Second, higher NIHSS scores in LAA‐related stroke have been correlated with lower clot burden scores (Tan et al. 2009; Sillanpaa et al. 2012). In large vessel occlusions with low clot burden, elevated endogenous plasminogen activity on admission may enhance susceptibility to thrombolysis (Szegedi et al. 2021), thereby reducing the relative advantage of antiplatelet therapy. In summary, the baseline NIHSS score serves as an indirect marker of both clot burden and collateral capacity‐two pivotal determinants of DAPT responsiveness in LAA stroke.

Regarding safety endpoints, patients receiving DAPT in both subgroups exhibited lower rates of SICH and other bleeding events, a profile consistent with previous reports (Chen et al. 2023; Fei et al. 2025). Therefore, DAPT safely improved functional outcomes in patients with LAA, without increasing the risk of hemorrhagic complications.

Our study has several limitations. First, the most significant limitation of our study is the reliance on the IPTW sensitivity analysis for the primary conclusion. While IPTW is a robust method to control for confounding in observational settings, it cannot fully replace the statistical power of a large, randomized controlled trial. The small sample size, particularly in the LAA subgroups, means that the non‐significant results in the primary analysis may represent a Type II error. Therefore, our findings should be considered exploratory and hypothesis‐generating, mandating validation in a dedicated, adequately powered prospective cohort or trial. Second, lack of blinded adjudication of LAA may introduce potential selection bias. Furthermore, a lack of white matter evaluation in the study may limit the interpretation about significant interaction between LAA and treatment efficacy based on metabolic syndrome. Third, the absence of repeated vascular imaging assessments limited our ability to confirm whether the presumed benefit of DAPT in preventing END was mediated through reducing reocclusion of the responsible vessel. Fourth, our findings are specifically applicable to minor stroke patients with LAA and cannot be generalized to those with large vessel occlusion, a subgroup of greater clinical urgency and therapeutic complexity. Fifth, constrained by the design of the ARAMIS trial, no patients received intravenous tenecteplase. Consequently, the efficacy of DAPT relative to tenecteplase in this specific population remains an open question worthy of future investigation. In summary, these limitations necessitate a cautious interpretation of our results, which should be validated in future studies with larger sample sizes and more comprehensive imaging protocols.

In conclusion, this study suggested that neurological deficit and LAA may affect efficacy of DAPT versus alteplase in acute minor nondisabling stroke. Among patients with milder neurologic deficit (NIHSS score 0–3) attributable to LAA, DAPT with clopidogrel and aspirin is associated with a numerically higher likelihood of excellent functional outcome at 90 days with a favorable safety profile, compared to intravenous alteplase. It is worth exploring efficacy of DAPT versus intravenous thrombolysis in this target population based on a large cohort, especially with tenecteplase.

## Author Contributions

**Yu Cui**: Writing – original draft, Writing – review and editing, formal analysis. **Zi‐Ang Li**: formal analysis, writing – original draft. **Chao He**: investigation. **Hui‐Sheng Chen**: conceptualization, writing – review and editing, supervision.

## Funding

This study was supported by grants from Self‐initiated Research Project of General Hospital of the Northern Theater Command (ZZKY2024014) and the Xingliao Talent Program of Liaoning Province (XLYC2403195). The funders had no role in the design and conduct of the study; collection, management, analysis, and interpretation of the data; preparation, review, or approval of the manuscript; and decision to submit the manuscript for publication.

## Ethics Statement

The study protocol was approved by the Ethics Committee of the General Hospital of Northern Theater Command [K(2018)22‐1] and other participating hospitals in accordance with the Declaration of Helsinki and International Ethical Guidelines for Human Biomedical Research. Signed informed consent was obtained from patients or their legally authorized representatives.

## Conflicts of Interest

The authors declare no conflicts of interest.

## Supporting information




**Supplementary Materials**: brb371813‐sup‐0001‐SuppMat.docx

## Data Availability

The datasets analyzed during the study are available upon reasonable request from the corresponding author.
