# Supplementary material for: NIHSS and Large Artery Atherosclerosis Affect Efficacy of Dual Antiplatelet Therapy Versus Alteplase for Minor Stroke
Source: Brain Behav. 2026 Oct 5;16(10):e71813. doi: 10.1002/brb3.71813 (PMC13639244; doi:10.1002/brb3.71813)
Supplement: Supplementary file 1 [file BRB3-16-e71813-s001.docx]

**Supplemental Materials for “Neurological Deficit Severity and Large Artery Atherosclerosis Affect Efficacy of Dual Antiplatelet Therapy versus Alteplase for Minor Stroke: Secondary Analysis of ARAMIS Trial”**

**Contents**

[Table S1. Baseline Characteristics of Patients Included and Excluded.	2](#_Toc14531)

[Table S2. Baseline Characteristics of Patients Between Subgroups.	3](#_Toc19331)

[Table S3. Baseline Characteristics Between Treatments in the NIHSS 0-3 & LAA Subgroup.	5](#_Toc4104)

[Table S4. Baseline Characteristics Between Treatments in the NIHSS 0-3 & Non-LAA Subgroup.	6](#_Toc8864)

[Table S5. Baseline Characteristics Between Treatments in the NIHSS 4-5 & LAA Subgroup.	7](#_Toc1810)

[Table S6. Baseline Characteristics Between Treatments in the NIHSS 4-5 & Non-LAA Subgroup.	8](#_Toc3839)

[Table S7. Inverse Probability of Treatment Weighting (IPTW) Characteristics.	9](#_Toc31136)

[Table S8. Baseline Characteristics of Patients with NIHSS 0-3 Between Subgroups.	10](#_Toc3062)

[Table S9. Baseline Characteristics of Patients NIHSS 4-5 Between Subgroups.	11](#_Toc4600)

# **Table S1. Baseline Characteristics of Patients Included and Excluded.**

|  | **Included (N=478)** | **Excluded (N=245)** | ***P* Value** |
| --- | --- | --- | --- |
| Age, y | 63 (56-70) | 66 (58-74) | 0.01 |
| Sex (F) | 147 (30.8) | 77 (31.4) | 0.85 |
| Current smoker | 165 (34.5) | 77 (31.4) | 0.70 |
| Current drinker ^a^ | 86 (18.0) | 30 (12.2) | 0.13 |
| Comorbidities ^b^ | | | |
| Hypertension | 260 (54.4) | 121 (49.4) | 0.20 |
| Diabetes | 128 (26.8) | 59 (24.1) | 0.43 |
| Previous stroke ^c^ | 102 (21.3) | 58 (23.7) | 0.47 |
| Previous transient ischemic attack | 3 (0.6) | 3 (1.2) | 0.40 |
| Blood pressure at randomization, mmHg | | | |
| Systolic | 153 (140-165) | 146 (136-161) | 0.01 |
| Diastolic | 88 (80-95) | 88 (80-96) | 0.75 |
| FBG at randomization, mmol/L | 6.42 (5.51-8.32) | 6.10 (5.21-7.54) | 0.23 |
| NIHSS score at randomization ^d^ | 2 (1-2) | 2 (1-3) | 0.23 |
| Estimated premorbid function (mRS score) ^e^ | | | |
| No symptoms (score, 0) | 360 (75.3) | 174 (71.0) | 0.21 |
| Symptoms without any disability (score, 1) | 118 (24.7) | 71 (29.0) |  |
| OTT, min | 179 (130-225) | 187 (131-230) | 0.51 |
| Duration of hospitalization, d | 8 (6-10) | 9 (7-11) | 0.02 |
| Presumed stroke etiology ^f^ | | | |
| Large artery atherosclerosis | 102 (21.3) | 1/244 (0.4) | <0.001 |
| Cardioembolic | 1 (0.2) | 1/244 (0.4) |  |
| Small artery occlusion | 165 (34.5) | 1/244 (0.4) |  |
| Other determined cause | 5 (1.0) | 0/244 (0.0) |  |
| Undetermined cause | 205 (42.9) | 242/244 (99.2) |  |
| Location of responsible vessel | | | |
| Anterior circulation | 358 (74.9) | 208 (84.9) | 0.01 |
| Posterior circulation | 116 (24.3) | 37 (15.1) |  |
| Anterior and posterior circulation | 4 (0.8) | 0 (0.0) |  |

The data was shown with median (interquartile range) or number (percentage). Abbreviation: FBG, fasting blood glucose; mRS, modified Rankin Scale; NIHSS, National Institute of Health Stroke Scale; OTT, time from onset of symptom to intravenous thrombolysis or dual antiplatelet therapy.

^a^ Defined as consuming alcohol at least once a week within 1 year prior to the onset of the disease.

^b^ The comorbidities were based on the patient or family report.

^c^ Previous stroke included ischemic and hemorrhagic stroke.

^d^ NIHSS scores range from 0 to 42, with higher scores indicating more severe neurological deficit.

^e^ Scores on the mRS of functional disability range from 0 (no symptoms) to 6 (death).

^f^ The presumed stroke etiology was classified according to the TOAST using clinical findings, brain imaging, and laboratory test results. Other causes included nonatherosclerotic vasculopathies, hypercoagulable states, and hematologic disorder.

^g^ The classification was defined according to the anatomical location of responsible vessel based on the patient’s clinical presentation and neuroimaging, which refers to the clinical features of the OCSP classification system.

# **Table S2. Baseline Characteristics of Patients Between Subgroups.**

|  | **NIHSS 0-3 & LAA**  **(N=84)** | **NIHSS 0-3 & Non-LAA**  **(N=304)** | **NIHSS 4-5 & LAA**  **(N=18)** | **NIHSS 4-5 & Non-LAA**  **(N=72)** | ***P* Value** |
| --- | --- | --- | --- | --- | --- |
| DAPT group | 37 (44.0) | 142 (46.7) | 6 (33.3) | 24 (33.3) | 0.17 |
| Age, y | 65 (60-71) | 63 (55-69) | 64 (59-69) | 64 (57-71) | 0.17 |
| Sex (F) | 21 (25.0) | 100 (32.9) | 1 (5.6) | 25 (34.7) | 0.049 |
| Current smoker | 32 (38.1) | 100 (32.9) | 8 (44.4) | 25 (34.7) | 0.74 |
| Current drinker ^a^ | 13 (24.5) | 47 (15.5) | 13 (24.5) | 47 (15.5) | 0.02 |
| Comorbidities ^b^ | | | | | |
| Hypertension | 52 (61.9) | 156 (51.3) | 10 (55.6) | 42 (58.3) | 0.32 |
| Diabetes | 28 (33.3) | 68 (22.4) | 6 (33.3) | 26 (36.1) | 0.04 |
| Previous stroke ^c^ | 17 (20.2) | 69 (22.7) | 2 (11.1) | 14 (19.4) | 0.64 |
| Previous transient ischemic attack | 1 (1.2) | 2 (0.7) | 0 (0.0) | 0 (0.0) | 0.80 |
| Blood pressure at randomization, mmHg | | | | | |
| Systolic | 153 (136-162) | 150 (139-165) | 155 (144-166) | 158 (146-171) | 0.06 |
| Diastolic | 83 (80-93) | 88 (80-95) | 90 (79-98) | 88 (81-96) | 0.38 |
| FBG at randomization, mmol/L | 6.60 (5.63-8.60) | 6.40 (5.44-7.81) | 5.90 (4.92-6.64) | 6.94 (5.70-10.90) | 0.03 |
| NIHSS score at randomization ^d^ | 2 (1-2) | 2 (1-2) | 4 (4-5) | 4 (4-5) | <0.001 |
| Estimated premorbid function (mRS score) ^e^ | | | | | |
| No symptoms (score, 0) | 65 (77.4) | 233 (76.6) | 13 (72.2) | 49 (68.1) | 0.46 |
| Symptoms without any disability (score, 1) | 19 (22.6) | 71 (23.4) | 5 (27.8) | 23 (31.9) |  |
| OTT, min | 171 (130-223) | 180 (129-231) | 182 (130-220) | 180 (126-205) | 0.73 |
| Duration of hospitalization, d | 9 (6-11) | 7 (6-10) | 8 (7-10) | 9 (7-11) | 0.01 |
| Presumed stroke etiology ^f^ | | | | | |
| Large artery atherosclerosis | 84 (100.0) | 0 (0.0) | 18 (100.0) | 0 (0.0) | <0.001 |
| Cardioembolic | 0 (0.0) | 1 (0.3) | 0 (0.0) | 0 (0.0) |  |
| Small artery occlusion | 0 (0.0) | 138 (45.4) | 0 (0.0) | 27 (37.5) |  |
| Other determined cause | 0 (0.0) | 5 (1.6) | 0 (0.0) | 0 (0.0) |  |
| Undetermined cause | 0 (0.0) | 160 (52.6) | 0 (0.0) | 45 (62.5) |  |
| Location of responsible vessel ^g^ | | | | | |
| Anterior circulation | 63 (75.0) | 227 (74.7) | 13 (72.2) | 55 (76.4) | 0.87 |
| Posterior circulation | 21 (25.0) | 73 (24.0) | 5 (27.8) | 17 (23.6) |  |
| Anterior and posterior circulation | 0 (0.0) | 4 (1.3) | 0 (0.0) | 0 (0.0) |  |

The data was shown with median (interquartile range) or number (percentage). Abbreviation: FBG, fasting blood glucose; mRS, modified Rankin Scale; NIHSS, National Institute of Health Stroke Scale; OTT, time from onset of symptom to intravenous thrombolysis or dual antiplatelet therapy.

^a^ Defined as consuming alcohol at least once a week within 1 year prior to the onset of the disease.

^b^ The comorbidities were based on the patient or family report.

^c^ Previous stroke included ischemic and hemorrhagic stroke.

^d^ NIHSS scores range from 0 to 42, with higher scores indicating more severe neurological deficit.

^e^ Scores on the mRS of functional disability range from 0 (no symptoms) to 6 (death).

^f^ The presumed stroke etiology was classified according to the TOAST using clinical findings, brain imaging, and laboratory test results. Other causes included nonatherosclerotic vasculopathies, hypercoagulable states, and hematologic disorder.

^g^ The classification was defined according to the anatomical location of responsible vessel based on the patient’s clinical presentation and neuroimaging, which refers to the clinical features of the OCSP classification system.

# **Table S3. Baseline Characteristics Between Treatments in the NIHSS 0-3 & LAA Subgroup.**

|  | **DAPT (N=37)** | **Alteplase (N=47)** | ***P* Value** |
| --- | --- | --- | --- |
| Age, y | 65 (60-72) | 65 (60-69) | 0.82 |
| Sex (F) | 10 (27.0) | 11 (23.4) | 0.70 |
| Current smoker | 14 (37.8) | 18 (38.3) | 0.71 |
| Current drinker ^a^ | 9 (24.3) | 11 (23.4) | 0.52 |
| Comorbidities ^b^ | | | |
| Hypertension | 25 (67.6) | 27 (57.4) | 0.34 |
| Diabetes | 11 (29.7) | 17 (36.2) | 0.53 |
| Previous stroke ^c^ | 9 (24.3) | 8 (17.0) | 0.41 |
| Previous transient ischemic attack | 1 (2.7) | 0 (0.0) | 0.26 |
| Blood pressure at randomization, mmHg | | | |
| Systolic | 142 (130-161) | 156 (146-164) | 0.08 |
| Diastolic | 83 (82-90) | 86 (79-100) | 0.31 |
| FBG at randomization, mmol/L | 6.18 (5.35-8.35) | 7.05 (5.74-9.14) | 0.25 |
| NIHSS score at randomization ^d^ | 1 (1-2) | 2 (1-2) | 0.047 |
| Estimated premorbid function (mRS score) ^e^ | | | |
| No symptoms (score, 0) | 27 (73.0) | 38 (80.9) | 0.39 |
| Symptoms without any disability (score, 1) | 10 (27.0) | 9 (19.1) |  |
| OTT, min | 167 (124-233) | 175 (144-203) | 0.96 |
| Duration of hospitalization, d | 10 (7-12) | 8 (6-10) | 0.10 |
| Presumed stroke etiology ^f^ | | | |
| Large artery atherosclerosis | 37 (100.0) | 47 (100.0) | >0.99 |
| Cardioembolic | 0 (0.0) | 0 (0.0) |  |
| Small artery occlusion | 0 (0.0) | 0 (0.0) |  |
| Other determined cause | 0 (0.0) | 0 (0.0) |  |
| Undetermined cause | 0 (0.0) | 0 (0.0) |  |
| Location of responsible vessel ^g^ | | | |
| Anterior circulation | 26 (70.3) | 37 (78.7) | 0.37 |
| Posterior circulation | 11 (29.7) | 10 (21.3) |  |
| Anterior and posterior circulation | 0 (0.0) | 0 (0.0) |  |

The data was shown with median (interquartile range) or number (percentage). Abbreviation: FBG, fasting blood glucose; mRS, modified Rankin Scale; NIHSS, National Institute of Health Stroke Scale; OTT, time from onset of symptom to intravenous thrombolysis or dual antiplatelet therapy.

^a^ Defined as consuming alcohol at least once a week within 1 year prior to the onset of the disease.

^b^ The comorbidities were based on the patient or family report.

^c^ Previous stroke included ischemic and hemorrhagic stroke.

^d^ NIHSS scores range from 0 to 42, with higher scores indicating more severe neurological deficit.

^e^ Scores on the mRS of functional disability range from 0 (no symptoms) to 6 (death).

^f^ The presumed stroke etiology was classified according to the TOAST using clinical findings, brain imaging, and laboratory test results. Other causes included nonatherosclerotic vasculopathies, hypercoagulable states, and hematologic disorder.

^g^ The classification was defined according to the anatomical location of responsible vessel based on the patient’s clinical presentation and neuroimaging, which refers to the clinical features of the OCSP classification system.

# **Table S4. Baseline Characteristics Between Treatments in the NIHSS 0-3 & Non-LAA Subgroup.**

|  | **DAPT (N=142)** | **Alteplase (N=162)** | ***P* Value** |
| --- | --- | --- | --- |
| Age, y | 64 (57-71) | 62 (55-67) | 0.02 |
| Sex (F) | 43 (30.3) | 57 (35.2) | 0.36 |
| Current smoker | 42 (29.6) | 58 (35.8) | 0.48 |
| Current drinker ^a^ | 23 (16.2) | 24 (14.8) | 0.80 |
| Comorbidities ^b^ | | | |
| Hypertension | 77 (54.2) | 79 (48.8) | 0.34 |
| Diabetes | 30 (21.1) | 38 (23.5) | 0.63 |
| Previous stroke ^c^ | 38 (26.8) | 31 (19.1) | 0.11 |
| Previous transient ischemic attack | 1 (0.7) | 1 (0.6) | 0.93 |
| Blood pressure at randomization, mmHg | | | |
| Systolic | 148 (137-163) | 152 (142-165) | 0.06 |
| Diastolic | 89 (80-95) | 87 (80-95) | 0.63 |
| FBG at randomization, mmol/L | 6.39 (5.41-7.98) | 6.41 (5.50-7.80) | 0.92 |
| NIHSS score at randomization ^d^ | 2 (1-2) | 2 (1-3) | 0.02 |
| Estimated premorbid function (mRS score) ^e^ | | | |
| No symptoms (score, 0) | 107 (75.4) | 126 (77.8) | 0.62 |
| Symptoms without any disability (score, 1) | 35 (24.6) | 36 (22.2) |  |
| OTT, min | 187 (132-239) | 168 (128-226) | 0.14 |
| Duration of hospitalization, d | 7 (6-10) | 7 (6-10) | 0.89 |
| Presumed stroke etiology ^f^ | | | |
| Large artery atherosclerosis | 0 (0.0) | 0 (0.0) | 0.58 |
| Cardioembolic | 1 (0.7) | 0 (0.0) |  |
| Small artery occlusion | 68 (47.9) | 70 (43.2) |  |
| Other determined cause | 2 (1.4) | 3 (1.9) |  |
| Undetermined cause | 71 (50.0) | 89 (54.9) |  |
| Location of responsible vessel ^g^ | | | |
| Anterior circulation | 107 (75.4) | 120 (74.1) | 0.95 |
| Posterior circulation | 33 (23.2) | 40 (24.7) |  |
| Anterior and posterior circulation | 2 (1.4) | 2 (1.2) |  |

The data was shown with median (interquartile range) or number (percentage). Abbreviation: FBG, fasting blood glucose; mRS, modified Rankin Scale; NIHSS, National Institute of Health Stroke Scale; OTT, time from onset of symptom to intravenous thrombolysis or dual antiplatelet therapy.

^a^ Defined as consuming alcohol at least once a week within 1 year prior to the onset of the disease.

^b^ The comorbidities were based on the patient or family report.

^c^ Previous stroke included ischemic and hemorrhagic stroke.

^d^ NIHSS scores range from 0 to 42, with higher scores indicating more severe neurological deficit.

^e^ Scores on the mRS of functional disability range from 0 (no symptoms) to 6 (death).

^f^ The presumed stroke etiology was classified according to the TOAST using clinical findings, brain imaging, and laboratory test results. Other causes included nonatherosclerotic vasculopathies, hypercoagulable states, and hematologic disorder.

^g^ The classification was defined according to the anatomical location of responsible vessel based on the patient’s clinical presentation and neuroimaging, which refers to the clinical features of the OCSP classification system.

# **Table S5. Baseline Characteristics Between Treatments in the NIHSS 4-5 & LAA Subgroup.**

|  | **DAPT (N=6)** | **Alteplase (N=12)** | ***P* Value** |
| --- | --- | --- | --- |
| Age, y | 71 (61-81) | 63 (57-68) | 0.10 |
| Sex (F) | 1 (16.7) | 0 (0.0) | 0.15 |
| Current smoker | 2 (33.3) | 6 (50.0) | 0.32 |
| Current drinker ^a^ | 1 (16.7) | 4 (33.3) | 0.22 |
| Comorbidities ^b^ | | | |
| Hypertension | 4 (66.7) | 6 (50.0) | 0.50 |
| Diabetes | 4 (66.7) | 2 (16.7) | 0.03 |
| Previous stroke ^c^ | 0 (0.0) | 2 (16.7) | 0.29 |
| Previous transient ischemic attack | 0 (0.0) | 0 (0.0) | >0.99 |
| Blood pressure at randomization, mmHg | | | |
| Systolic | 149 (125-157) | 156 (147-174) | 0.13 |
| Diastolic | 84 (78-91) | 92 (82-99) | 0.15 |
| FBG at randomization, mmol/L | 6.47 (4.92-8.05) | 5.75 (4.86-6.32) | 0.51 |
| NIHSS score at randomization ^d^ | 4 (4-4) | 4 (4-5) | 0.62 |
| Estimated premorbid function (mRS score) ^e^ | | | |
| No symptoms (score, 0) | 4 (66.7) | 9 (75.0) | 0.71 |
| Symptoms without any disability (score, 1) | 2 (33.3) | 3 (25.0) |  |
| OTT, min | 203 (158-220) | 154 (111-229) | 0.44 |
| Duration of hospitalization, d | 10 (9-16) | 8 (6-9) | 0.01 |
| Presumed stroke etiology ^f^ | | | |
| Large artery atherosclerosis | 6 (100.0) | 12 (100.0) | >0.99 |
| Cardioembolic | 0 (0.0) | 0 (0.0) |  |
| Small artery occlusion | 0 (0.0) | 0 (0.0) |  |
| Other determined cause | 0 (0.0) | 0 (0.0) |  |
| Undetermined cause | 0 (0.0) | 0 (0.0) |  |
| Location of responsible vessel ^g^ | | | |
| Anterior circulation | 5 (83.3) | 8 (66.7) | 0.46 |
| Posterior circulation | 1 (16.7) | 4 (33.3) |  |
| Anterior and posterior circulation | 0 (0.0) | 0 (0.0) |  |

The data was shown with median (interquartile range) or number (percentage). Abbreviation: FBG, fasting blood glucose; mRS, modified Rankin Scale; NIHSS, National Institute of Health Stroke Scale; OTT, time from onset of symptom to intravenous thrombolysis or dual antiplatelet therapy.

^a^ Defined as consuming alcohol at least once a week within 1 year prior to the onset of the disease.

^b^ The comorbidities were based on the patient or family report.

^c^ Previous stroke included ischemic and hemorrhagic stroke.

^d^ NIHSS scores range from 0 to 42, with higher scores indicating more severe neurological deficit.

^e^ Scores on the mRS of functional disability range from 0 (no symptoms) to 6 (death).

^f^ The presumed stroke etiology was classified according to the TOAST using clinical findings, brain imaging, and laboratory test results. Other causes included nonatherosclerotic vasculopathies, hypercoagulable states, and hematologic disorder.

^g^ The classification was defined according to the anatomical location of responsible vessel based on the patient’s clinical presentation and neuroimaging, which refers to the clinical features of the OCSP classification system.

# **Table S6. Baseline Characteristics Between Treatments in the NIHSS 4-5 & Non-LAA Subgroup.**

|  | **DAPT (N=24)** | **Alteplase (N=48)** | ***P* Value** |
| --- | --- | --- | --- |
| Age, y | 67 (57-75) | 63 (57-71) | 0.44 |
| Sex (F) | 9 (37.5) | 16 (33.3) | 0.73 |
| Current smoker | 5 (20.8) | 20 (41.7) | 0.04 |
| Current drinker ^a^ | 2 (8.3) | 12 (25.0) | 0.26 |
| Comorbidities ^b^ | | | |
| Hypertension | 18 (75.0) | 24 (50.0) | 0.04 |
| Diabetes | 9 (37.5) | 17 (35.4) | 0.86 |
| Previous stroke ^c^ | 4 (16.7) | 10 (20.8) | 0.67 |
| Previous transient ischemic attack | 0 (0.0) | 0 (0.0) | >0.99 |
| Blood pressure at randomization, mmHg | | | |
| Systolic | 154 (144-170) | 160 (146-171) | 0.25 |
| Diastolic | 89 (81-96) | 88 (80-97) | 0.96 |
| FBG at randomization, mmol/L | 7.74 (5.69-13.10) | 6.30(5.63-8.09) | 0.06 |
| NIHSS score at randomization ^d^ | 4 (4-5) | 4 (4-5) | 0.40 |
| Estimated premorbid function (mRS score) ^e^ | | | |
| No symptoms (score, 0) | 14 (58.3) | 35 (72.9) | 0.21 |
| Symptoms without any disability (score, 1) | 10 (41.7) | 13 (27.1) |  |
| OTT, min | 185 (131-216) | 180 (124-200) | 0.55 |
| Duration of hospitalization, d | 9 (8-12) | 9 (7-11) | 0.29 |
| Presumed stroke etiology ^f^ | | | |
| Large artery atherosclerosis | 0 (0.0) | 0 (0.0) | 0.12 |
| Cardioembolic | 0 (0.0) | 0 (0.0) |  |
| Small artery occlusion | 6 (25.0) | 21 (43.8) |  |
| Other determined cause | 0 (0.0) | 0 (0.0) |  |
| Undetermined cause | 18 (75.0) | 27 (56.3) |  |
| Location of responsible vessel ^g^ | | | |
| Anterior circulation | 19 (79.2) | 36 (75.0) | 0.70 |
| Posterior circulation | 5 (20.8) | 12 (25.0) |  |
| Anterior and posterior circulation | 0 (0.0) | 0 (0.0) |  |

The data was shown with median (interquartile range) or number (percentage). Abbreviation: FBG, fasting blood glucose; mRS, modified Rankin Scale; NIHSS, National Institute of Health Stroke Scale; OTT, time from onset of symptom to intravenous thrombolysis or dual antiplatelet therapy.

^a^ Defined as consuming alcohol at least once a week within 1 year prior to the onset of the disease.

^b^ The comorbidities were based on the patient or family report.

^c^ Previous stroke included ischemic and hemorrhagic stroke.

^d^ NIHSS scores range from 0 to 42, with higher scores indicating more severe neurological deficit.

^e^ Scores on the mRS of functional disability range from 0 (no symptoms) to 6 (death).

^f^ The presumed stroke etiology was classified according to the TOAST using clinical findings, brain imaging, and laboratory test results. Other causes included nonatherosclerotic vasculopathies, hypercoagulable states, and hematologic disorder.

^g^ The classification was defined according to the anatomical location of responsible vessel based on the patient’s clinical presentation and neuroimaging, which refers to the clinical features of the OCSP classification system.

# **Table S7. Inverse Probability of Treatment Weighting (IPTW) Characteristics.**

| **Weight Characteristics** | **NIHSS score of 0-3** | | | | **NIHSS score of 4-5** | | | |
| --- | --- | --- | --- | --- | --- | --- | --- | --- |
|  | **LAA subgroup**  **(DAPT vs. Alteplase)** | | **Non-LAA subgroup**  **(DAPT vs. Alteplase)** | | **LAA subgroup**  **(DAPT vs. Alteplase)** | | **Non-LAA subgroup**  **(DAPT vs. Alteplase)** | |
| Mean weight | 2.02 | | 1.23 | | 1.59 | | 1.90 | |
| Min weight | 1.12 | | 0.25 | | 1.00 | | 1.01 | |
| Max weight | 7.62 | | 3.72 | | 6.08 | | 4.91 | |
| Effective sample size after weighting | 170 | | 375 | | 29 | | 136 | |
| **Standardized Mean Differences** | **Before IPTW** | **After IPTW** | **Before IPTW** | **After IPTW** | **Before IPTW** | **After IPTW** | **Before IPTW** | **After IPTW** |
| Age | 0.083 | 0.088 | 0.286 | 0.317 | 1.038 | 0.359 | 0.229 | 0.062 |
| Sex (F) | 0.083 | 0.019 | 0.105 | 0.124 | 0.062 | 0.406 | 0.087 | 0.042 |
| Current smoker | 0.245 | 0.259 | 0.181 | 0.184 | 1.000 | 1.229 | 0.675 | 0.663 |
| Current drinker | 0.328 | 0.274 | 0.116 | 0.156 | 1.201 | 1.529 | 0.523 | 0.530 |
| Hypertension | 0.210 | 0.178 | 0.109 | 0.084 | 0.343 | 0.467 | 0.535 | 1.577 |
| Diabetes | 0.137 | 0.033 | 0.056 | 0.024 | 1.177 | 0.425 | 0.043 | 0.095 |
| Previous stroke | 0.181 | 0.089 | 0.182 | 0.190 | 0.632 | 0.566 | 0.107 | 0.104 |
| Previous transient ischemic attack | 0.236 | 0.256 | 0.011 | 0.019 | <0.001 | <0.001 | <0.001 | <0.001 |
| Systolic blood pressure at randomization | 0.428 | 0.014 | 0.218 | 0.177 | 0.881 | 0.386 | 0.310 | 0.184 |
| Diastolic blood pressure at randomization | 0.373 | 0.207 | 0.051 | 0.042 | 0.575 | 0.536 | 0.009 | 0.038 |
| Fasting blood glucose at randomization | 0.214 | 0.206 | 0.125 | 0.101 | 0.595 | 0.436 | 0.223 | 0.119 |
| NIHSS score at randomization | 0.466 | 0.052 | 0.291 | 0.245 | 0.369 | 0.443 | 0.210 | 0.002 |
| Estimated premorbid function | 0.188 | 0.274 | 0.057 | 0.083 | 0.184 | 0.511 | 0.311 | 0.252 |
| OTT | 0.030 | 0.073 | 0.143 | 0.205 | 0.490 | 0.056 | 0.137 | 0.077 |
| Duration of hospitalization, d | 0.081 | 0.233 | 0.031 | 0.009 | 1.493 | 2.311 | 0.203 | 0.136 |
| Presumed stroke etiology | 0.209 | 0.237 | 0.158 | 0.211 | 0.426 | 0.395 | 0.403 | 0.307 |
| Location of responsible vessel | 0.195 | 0.030 | 0.036 | 0.044 | 0.392 | 0.224 | 0.099 | 0.087 |

Abbreviation: NIHSS, National Institute of Health Stroke Scale; OTT, time from onset of symptom to intravenous thrombolysis or dual antiplatelet.

# **Table S8. Baseline Characteristics of Patients with NIHSS 0-3 Between Subgroups.**

|  | **LAA (N=84)** | **Non-LAA (N=304)** | ***P* Value** |
| --- | --- | --- | --- |
| Age | 65 (60-71) | 63 (55-69) | 0.06 |
| Sex (F) | 21 (25.0) | 100 (32.9) | 0.17 |
| Current smoker | 32 (38.1) | 100 (32.9) | 0.57 |
| Current drinker ^a^ | 13 (24.5) | 47 (15.5) | 0.29 |
| Comorbidities ^b^ | | | |
| Hypertension | 52 (61.9) | 156 (51.3) | 0.09 |
| Diabetes | 28 (33.3) | 68 (22.4) | 0.04 |
| Previous stroke ^c^ | 17 (20.2) | 69 (22.7) | 0.63 |
| Previous transient ischemic attack | 1 (1.2) | 2 (0.7) | 0.62 |
| Blood pressure at randomization, mmHg | | | |
| Systolic | 153 (136-162) | 150 (139-165) | 0.86 |
| Diastolic | 83 (80-93) | 88 (80-95) | 0.13 |
| FBG at randomization, mmol/L | 6.60 (5.63-8.60) | 6.40 (5.44-7.81) | 0.32 |
| NIHSS score at randomization ^d^ | 2 (1-2) | 2 (1-2) | 0.94 |
| Estimated premorbid function (mRS score) ^e^ | | | |
| No symptoms (score, 0) | 65 (77.4) | 233 (76.6) | 0.89 |
| Symptoms without any disability (score, 1) | 19 (22.6) | 71 (23.4) |  |
| OTT, min | 171 (130-223) | 180 (129-231) | 0.69 |
| Duration of hospitalization, d | 9 (6-11) | 7 (6-10) | 0.01 |
| Presumed stroke etiology ^f^ | | | |
| Large artery atherosclerosis | 84 (100.0) | 0 (0.0) | <0.001 |
| Cardioembolic | 0 (0.0) | 1 (0.3) |  |
| Small artery occlusion | 0 (0.0) | 138 (45.4) |  |
| Other determined cause | 0 (0.0) | 5 (1.6) |  |
| Undetermined cause | 0 (0.0) | 160 (52.6) |  |
| Location of responsible vessel | | | |
| Anterior circulation | 63 (75.0) | 227 (74.7) | 0.57 |
| Posterior circulation | 21 (25.0) | 73 (24.0) |  |
| Anterior and posterior circulation | 0 (0.0) | 4 (1.3) |  |

The data was shown with median (interquartile range) or number (percentage). Abbreviation: FBG, fasting blood glucose; mRS, modified Rankin Scale; NIHSS, National Institute of Health Stroke Scale; OTT, time from onset of symptom to intravenous thrombolysis or dual antiplatelet therapy.

^a^ Defined as consuming alcohol at least once a week within 1 year prior to the onset of the disease.

^b^ The comorbidities were based on the patient or family report.

^c^ Previous stroke included ischemic and hemorrhagic stroke.

^d^ NIHSS scores range from 0 to 42, with higher scores indicating more severe neurological deficit.

^e^ Scores on the mRS of functional disability range from 0 (no symptoms) to 6 (death).

^f^ The presumed stroke etiology was classified according to the TOAST using clinical findings, brain imaging, and laboratory test results. Other causes included nonatherosclerotic vasculopathies, hypercoagulable states, and hematologic disorder.

^g^ The classification was defined according to the anatomical location of responsible vessel based on the patient’s clinical presentation and neuroimaging, which refers to the clinical features of the OCSP classification system.

# **Table S9. Baseline Characteristics of Patients NIHSS 4-5 Between Subgroups.**

|  | **LAA (N=18)** | **Non-LAA (N=72)** | ***P* Value** |
| --- | --- | --- | --- |
| Age | 64 (59-69) | 64 (57-71) | 0.91 |
| Sex (F) | 1 (5.6) | 25 (34.7) | 0.02 |
| Current smoker | 8 (44.4) | 25 (34.7) | 0.40 |
| Current drinker ^a^ | 13 (24.5) | 47 (15.5) | 0.18 |
| Comorbidities ^b^ | | | |
| Hypertension | 10 (55.6) | 42 (58.3) | 0.83 |
| Diabetes | 6 (33.3) | 26 (36.1) | 0.83 |
| Previous stroke ^c^ | 2 (11.1) | 14 (19.4) | 0.41 |
| Previous transient ischemic attack | 0 (0.0) | 0 (0.0) | >0.99 |
| Blood pressure at randomization, mmHg | | | |
| Systolic | 155 (144-166) | 158 (146-171) | 0.41 |
| Diastolic | 90 (79-98) | 88 (81-96) | 0.75 |
| FBG at randomization, mmol/L | 5.90 (4.92-6.64) | 6.94 (5.70-10.90) | 0.01 |
| NIHSS score at randomization ^d^ | 4 (4-5) | 4 (4-5) | 0.38 |
| Estimated premorbid function (mRS score) ^e^ | | | |
| No symptoms (score, 0) | 13 (72.2) | 49 (68.1) | 0.73 |
| Symptoms without any disability (score, 1) | 5 (27.8) | 23 (31.9) |  |
| OTT, min | 182 (130-220) | 180 (126-205) | 0.64 |
| Duration of hospitalization, d | 8 (7-10) | 9 (7-11) | 0.50 |
| Presumed stroke etiology ^f^ | | | |
| Large artery atherosclerosis | 18 (100.0) | 0 (0.0) | <0.001 |
| Cardioembolic | 0 (0.0) | 0 (0.0) |  |
| Small artery occlusion | 0 (0.0) | 27 (37.5) |  |
| Other determined cause | 0 (0.0) | 0 (0.0) |  |
| Undetermined cause | 0 (0.0) | 45 (62.5) |  |
| Location of responsible vessel ^g^ | | | |
| Anterior circulation | 13 (72.2) | 55 (76.4) | 0.71 |
| Posterior circulation | 5 (27.8) | 17 (23.6) |  |
| Anterior and posterior circulation | 0 (0.0) | 0 (0.0) |  |

The data was shown with median (interquartile range) or number (percentage). Abbreviation: FBG, fasting blood glucose; mRS, modified Rankin Scale; NIHSS, National Institute of Health Stroke Scale; OTT, time from onset of symptom to intravenous thrombolysis or dual antiplatelet therapy.

^a^ Defined as consuming alcohol at least once a week within 1 year prior to the onset of the disease.

^b^ The comorbidities were based on the patient or family report.

^c^ Previous stroke included ischemic and hemorrhagic stroke.

^d^ NIHSS scores range from 0 to 42, with higher scores indicating more severe neurological deficit.

^e^ Scores on the mRS of functional disability range from 0 (no symptoms) to 6 (death).

^f^ The presumed stroke etiology was classified according to the TOAST using clinical findings, brain imaging, and laboratory test results. Other causes included nonatherosclerotic vasculopathies, hypercoagulable states, and hematologic disorder.

^g^ The classification was defined according to the anatomical location of responsible vessel based on the patient’s clinical presentation and neuroimaging, which refers to the clinical features of the OCSP classification system.
